# Characterization and Expression Analysis of Genes from *Megalobrama amblycephala* Encoding Hemoglobins with Extracellular Microbicidal Activity

**DOI:** 10.3390/genes14101972

**Published:** 2023-10-22

**Authors:** Qijun Wang, Xiaoheng Zhao, Yunlong Liu, Juan Zheng, Hujun Cui, Haotong Wang, Houxu Ding, Hong Liu, Zhujin Ding

**Affiliations:** 1 Shaanxi Key Laboratory of Qinling Ecological Security, Shaanxi Institute of Zoology, Xi’an 710032, China; wangqijun@xab.ac.cn; 2 Co-Innovation Center of Jiangsu Marine Bio-Industry Technology, Jiangsu Key Laboratory of Marine Bioresources and Environment, Jiangsu Ocean University, Lianyungang 222005, China; zhaoxiaoheng@jou.edu.cn (X.Z.); lyl4912@163.com (Y.L.); cuihujun@jou.edu.cn (H.C.); wht011029@163.com (H.W.); 2021220108@jou.edu.cn (H.D.); 3 Jiangsu Key Laboratory of Marine Biotechnology, School of Marine Science and Fisheries, Jiangsu Ocean University, Lianyungang 222005, China; 4 Shaanxi Environmental Survey and Evaluation Center, Xi’an 710054, China; fox_82821@163.com; 5 College of Fisheries, Huazhong Agricultural University, Wuhan 430070, China; liuhong59@mail.hzau.edu.cn

**Keywords:** *Megalobrama amblycephala*, hemoglobin subunits, characterization, expression pattern, antimicrobial activity

## Abstract

Hemoglobin (Hb) usually comprises two α and two β subunits, forming a tetramer responsible for oxygen transportation and storage. Few studies have elucidated fish hemoglobin immune functions. *Megalobrama amblycephala* is a freshwater-cultured fish prevalent in China. We identified two *M. amblycephala* hemoglobin subunits and analyzed their expression patterns and antibacterial activities. The respective full-length cDNA sequences of the *M. amblycephala Hb α* (*MaHbα*) and *β* (*MaHbβ*) subunits were 588 and 603 bp, encoding 143 and 148 amino acids. MaHbα and MaHbβ were highly homologous to hemoglobins from other fish, displaying typical globin-like domains, most heme-binding sites, and tetramer interface regions highly conserved in teleosts. In phylogenetic analyses, the hemoglobin genes from *M. amblycephala* and other cypriniformes clustered into one branch, and those from other fishes and mammals clustered into other branches, revealing fish hemoglobin conservation. These *M. amblycephala Hb* subunits exhibit different expression patterns in various tissues and during development. *MaHbα* is mainly expressed in the blood and brain, while *MaHbβ* gene expression is highest in the muscle. *MaHbα* expression was detectable and abundant post-fertilization, with levels fluctuating during the developmental stages. *MaHbβ* expression began at 3 dph and gradually increased. Expression of both *M. amblycephala Hb* subunits was down-regulated in most examined tissues and time points post-*Aeromonas hydrophila* infection, which might be due to red blood cell (RBC) and hematopoietic organ damage. Synthetic MaHbα and MaHbβ peptides showed excellent antimicrobial activities, which could inhibit survival and growth in five aquatic pathogens. Two *M. amblycephala* hemoglobin subunits were identified, and their expression patterns and antibacterial activities were analyzed, thereby providing a basis for the understanding of evolution and functions of fish hemoglobins.

## 1. Introduction

Hemoglobin (Hb) is predominantly expressed in red blood cells (RBCs). Vertebrate hemoglobin is a globular protein with a tetrahedral structure composed of four globin protein subunits (two α and two β), each containing a prosthetic group called heme, allowing the binding of four oxygen (O_2_) molecules. Hemoglobin is primarily responsible for oxygen transport, and plays a particularly important role in facilitating the adaptation of fish to changing external environments and dissolved oxygen conditions. The oxygen affinity of hemoglobin is affected by the structural state of its two subunits. In oxygen-rich tissues, the subunit structure is relaxed, and oxygen binds to heme to form Hb-O_2_, allowing oxygen transport to tissues that require it for oxidative respiration. In hypoxic tissues, the subunits are in tense conformation, weakening their affinity for oxygen, resulting in the dissociation of Hb-O_2_ and the release of oxygen in tissues that require it. 

While hemoglobin is a well-known oxygen-carrying protein in the blood, other functions of hemoglobins have not been thoroughly described. Recent studies revealed that hemoglobin can exhibit considerable antibacterial effects [1,2,3]. Assays of a purified Hb fragment found that it could inhibit the proliferation of *Escherichia coli*, *Micrococcus luteus,* and *Listeria monocytogenes* [4]. When Hb is oxidized to methemoglobin, superoxide anions are produced by peroxide-like enzymes, and other toxic substances, such as hydroxyl radicals, which have sterilizing effects, are derived from these superoxide anions [5]. In addition, in response to bacterial invasion and the release of virulence factors, animals can oxidize host Hb, thereby producing a large number of reactive oxygen species and effectively killing pathogens [6]. The antimicrobial properties of mammalian hemoglobins have been well characterized; however, few studies have been conducted on fish [7].

The molecular organization of *α*- and *β-globin* genes has been studied in several teleost species, including Atlantic salmon [8], rainbow trout [9], zebrafish [10], carp [11], yellowtail [12], puffer fish [13], and medaka [14]. Characterization and functional analyses of *hemoglobins* from other aquatic animals, including blood clams [15,16] and ark shells [17], have also been performed. At present, limited study has been devoted to hemoglobin genes in aquatic animals. Thus, its gene characterization, roles in oxygen transport, iron ion metabolism, and immune function need to be further studied to better understand the regulation of hypoxia and disease resistance in aquatic animals.

Blunt snout bream (*Megalobrama amblycephala*) is an economically important fish species in China’s freshwater aquaculture system that is susceptible to bacterial septicemia caused by *Aeromonas hydrophila* infection. *A. hydrophila* is a Gram-negative bacterium that causes widespread infection in cultured fish, and a large-scale epidemic of septicemia caused by *A. hydrophila*-infection has led to significant economic losses. Although most studies have shown that blood cells in teleosts exert significant antibacterial effects, few studies have directly demonstrated that hemoglobin exerts an antibacterial immune function. Studies of fish hemoglobin participating in other vital physiological processes in vivo, such as innate immune homeostasis and signal transduction, should thus be conducted. In the present study, we identified and characterized two *M. amblycephala* hemoglobin subunits (*MaHbs*), an α subunit (*MaHbα*) and a β subunit (*MaHbβ*). We characterized these genes’ expression patterns and the antimicrobial activity of natural and synthesized MaHbs against the fish pathogen *A. hydrophila*, thereby laying a foundation for revealing the potential immune roles and mechanisms of MaHbs against *A. hydrophila* infection.

## 2. Materials and Methods

### 2.1. Ethics Statement

This study was approved by the Animal Care and Use Committee of Jiangsu Ocean University (protocol no. 2020-37, approval date: 1 September 2019). All animal procedures were performed according to the Guidelines for the Care and Use of Laboratory Animals in China.

### 2.2. Sample Collection

Adult *M. amblycephala* (400 ± 30 g), obtained from the Ezhou breeding base of Huazhong Agricultural University, were cultured in a recirculating aquaculture system for two weeks. To investigate the expression patterns of target genes in various healthy tissues, nine tissues were collected from six adult *M. amblycephala*, including liver, spleen, kidney, intestine, gill, brain, heart, muscle, and blood. Experimental fish were anesthetized using 100 mg/L MS-222 before dissection, and all samples were flash-frozen in liquid nitrogen for 24 h and then used for RNA extraction. 

Fertilized eggs were cultured in a recirculating aquaculture system at 25 ± 1 °C. To explore target gene expression patterns during early developmental stages, embryos at 0, 2, 6, 12, 19, 26, and 32 h post-fertilization (hpf), and fish larvae at 3, 5, and 15 days post-hatching (dph) were collected.

Bacterial challenge was conducted as previously described [18]. Briefly, 600 juvenile *M. amblycephala* (14.6 ± 0.6 g) were sorted into the control and challenge groups, which were intraperitoneally injected with 100 μL 0.6% saline solution or pathogenic *A. hydrophila* (1 × 10^7^ CFU/mL, half lethal dosage), respectively. Then, thirty experimental fish from each group were randomly dissected to collect the gill, spleen, kidney, and liver at 0, 4, 24, 72, and 120 h post-injection (hpi).

### 2.3. RNA Isolation and cDNA Synthesis

The above collected samples were homogenized in liquid nitrogen, and the total RNA was isolated by using TRIzol Reagent (TaKaRa, Dalian, China). The quality and concentration of the total RNA were detected using agarose gel electrophoresis and a NanoDrop 2000 (Thermo Scientific, Waltham, MA, USA), respectively. Then, 1 μg of the total RNA was used for first-strand cDNA synthesis using PrimeScript^®^ RT reagent Kit with gDNA Eraser (TaKaRa), which was further used for RT-qPCR analysis.

### 2.4. Identification of M. amblycephala Hemoglobin Genes

The coding regions of the *M. amblycephala Hbα* and *Hbβ* genes were obtained from the *M. amblycephala* genome database [19], which were verified using PCR amplification. To obtain the full-length cDNA sequences of the *MaHbα* and *MaHbβ* genes, 3′- and 5′- rapid-amplification of cDNA ends polymerase chain reactions (RACE-PCR) were conducted using a SMART™ RACE cDNA Amplification Kit (TaKaRa), and the primers are presented in Table 1. The RACE-PCR products were ligated with a pGEM^®^-T Easy Vector (Promega, Fitchburg, WI, USA) and transformed into trans5α competent cells, which were then sequenced in Beijing Genomics Institute (Wuhan, China). The full-length cDNA sequences of the *M. amblycephala Hbα* and *Hbβ* genes were assembled using the SeqMan version 7.1 software in the DNASTAR package. 

### 2.5. Sequence and Phylogenetic Analysis

The amino acid sequence of MaHbs was predicted using Open Reading Frame Finder on the NCBI website (http://www.ncbi.nlm.nih.gov/gorf/orfig.cgi; accessed on 20 August 2023). The amino acid composition, theoretical molecular weight, and isoelectric points of the protein-coding regions were analyzed using the ExPASy ProtParam website (https://web.expasy.org/protparam/; accessed on 20 August 2023). Conserved domains and heme-oxygen binding sites were predicted using the ExPASy Prosite database (http://prosite.expasy.org; accessed on 20 August 2023) and SMART program (http://smart.embl-heidelberg.de/; accessed on 20 August 2023). Predicted 3D protein structural models were established using the SWISSMODEL prediction algorithm (http://swissmodel.expasy.org; accessed on 20 August 2023). Multiple sequence alignments were performed using CLUSTAL (accession numbers are listed in Supplementary Table S1). The nucleotide sequences of various vertebrate hemoglobins (accession numbers are listed in Supplementary Tables S2 and S3) retrieved from GenBank were used for phylogenetic tree construction, which was performed using MEGA 11.0 software, using the neighbor-joining method [20].

### 2.6. Quantitative Real-Time PCR Analysis

The expression patterns of the *M. amblycephala Hbα* and *Hbβ* genes were detected using quantitative real-time PCR (RT-qPCR) as previously described [21]. Briefly, RT-qPCR was conducted in triplicate on a real-time PCR detection system (QIAGEN, Dusseldorf, Germany) using a SYBR^®^ Premix Ex Taq™ kit (TaKaRa). *18S rRNA* was selected as the internal reference gene using geNorm version 3.5 [22], with the primers presented in Table 1. The RT-qPCR specificity was assessed using melting curve analysis and direct sequencing of the RT-qPCR products. The amplification efficiency, threshold, and formula for the relative copies of the target genes were analyzed by using constructed double standard curves. Then, the relative expression levels of target genes were evaluated using the ratios of the relative copies of the target genes to *18S rRNA*.

### 2.7. Antimicrobial Activities of Synthesized MaHbα and MaHbβ Peptides

The MaHbα peptide (107–136, IIVVIGMLFPADFTPEVHVSVDKFFQCLAL) and MaHbβ peptide (116–146, AMKFGPSGFNADVQEAWQKFLSVVVSALCRQ) were synthesized by ChinaPeptides (QYAOBIO) co. Ltd. (Shanghai, China). The underlined sites are phosphorylation sites. These peptides incorporate N-terminal acetylation and C-terminal amidation modifications, and exhibit over 95% purity. 

To analyze the effects of the synthesized MaHbα and MaHbβ peptides on the growth of aquatic pathogens, including *A. hydrophila*, *Aeromonas sobria*, *Vibrio harveyi*, *Edwardsiella tarda*, and *Escherichia coli*, their antimicrobial activities were determined using liquid growth inhibition assay in a microtiter plate assay system as previously described with some modifications [23]. Briefly, the bacteria were cultured to an exponential phase in a liquid LB medium at 28 °C, and then diluted (1:100) with fresh LB medium. The diluted cultures were then supplemented with synthetic MaHbα and MaHbβ peptides to a final concentration of 30 μg/mL, with the normal LB medium as the control. These bacteria were incubated at 28 °C for 24 h. The microbial growth and bacterial density in the liquid LB medium were assessed by measuring the absorbance at 600 nm at different time points (3, 6, 9, 12, 18, and 24 h) post-incubation with synthetic MaHbα and MaHbβ peptides. These assays were conducted in triplicate.

### 2.8. Statistical Analyses

All data are presented as means ± SEM and statistical significance was assessed using one-way analysis of variance (ANOVA) using SPSS (version 17.0, Chicago, IL, USA), with *p* < 0.05 considered as a statistically significant difference.

## 3. Results

### 3.1. Identification and Characterization of MaHb Genes

**Figure 1:**
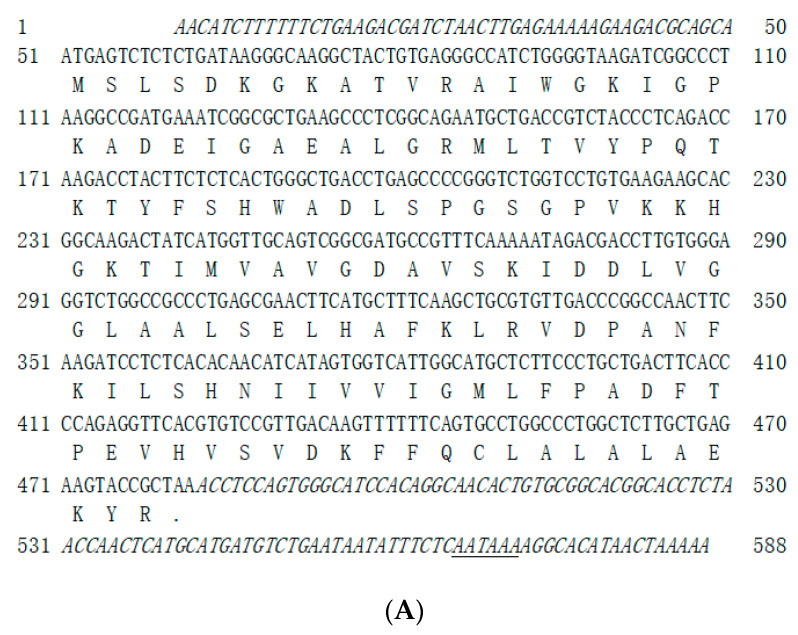

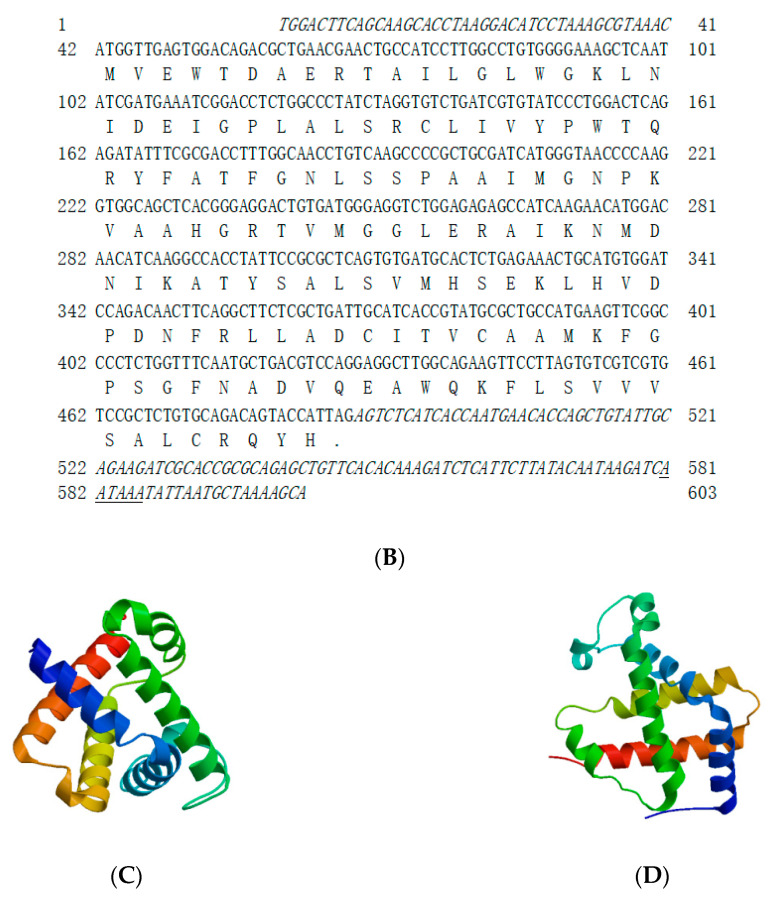
Nucleotide/amino acid sequence diagram and predicted tertiary structures of *Megalobrama amblycephala* hemoglobins α (**A**,**C**) and β (**B**,**D**) subunits.

In the present study, the complete cDNA sequences for the *MaHbα* and *MaHbβ* genes were identified and characterized, and have been deposited in GenBank with the respective accession numbers KP288030 and OR594269. The full-length cDNA sequences of the *MaHbα* and *MaHbβ* genes were 588 and 603 bp in length, and encoded 143 and 148 amino acids, with predicted molecular weights of 15.5 and 16.4 kDa, and theoretical isoelectric points of 9.02 and 7.70, respectively. MaHbα and MaHbβ possessed no signal peptide or transmembrane domain, and were consistent with sequences from other fish species (Figure 1). The respective aliphatic amino acid indices of MaHbα and MaHbβ were 100.28 and 91.01, and their respective grand averages of hydropathicity (GRAVY) were 0.182 and 0.095. The total numbers of negatively charged (Asp+Glu) and positively charged (Arg+Lys) residues in MaHbα were 14 and 17, and those in MaHbβ were 13 and 14, respectively. Both MaHbα and MaHbβ showed the typical structural characteristics of hemoglobins with a globin-like fold and plenty of α-helices in the predicted tertiary structures (Figure 1).

### 3.2. Multiple Sequence Alignment and Phylogenetic Analysis of MaHb Genes

**Figure 2:**
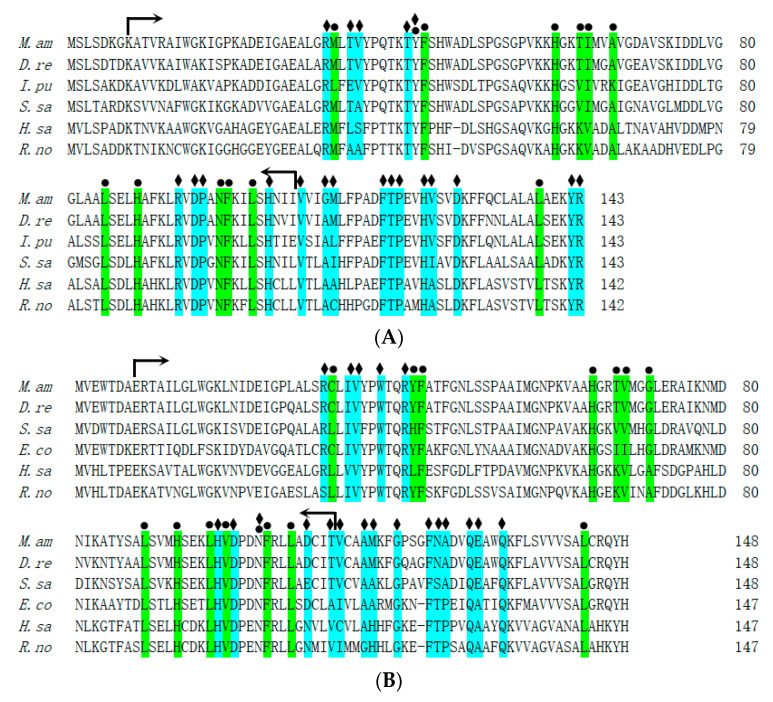
Multiple alignments of the amino acid sequences of hemoglobin α (**A**) and β (**B**) subunits. Notes: *M. am*, *M. amblycephala*; *D. re*, *Danio rerio*; *I. pu*, *Ictalurus punctatus*; *S. sa*, *Salmo salar*; *H. sa*, *Homo sapiens*; *R. no*, *Rattus norvegicus*; *E. co*, *Epinephelus coioides*. The green columns under filled circles represent heme-binding sites, the blue columns under filled diamonds represent the tetramer interface (polypeptide-binding sites), and the spans in between arrows represent globin-like domains. The accession numbers for these amino acid sequences are listed in Supplementary Table S1.

**Figure 3:**
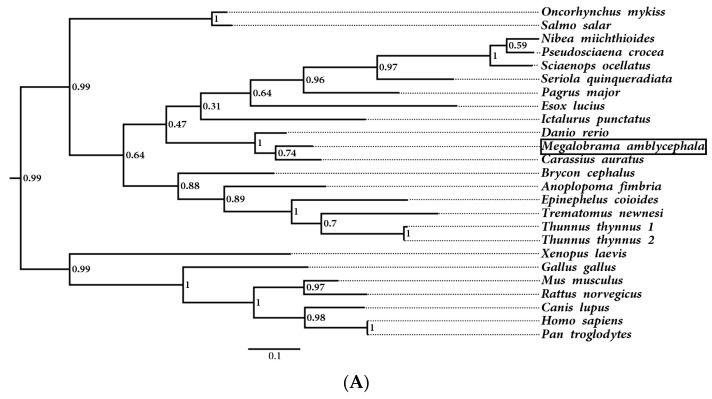

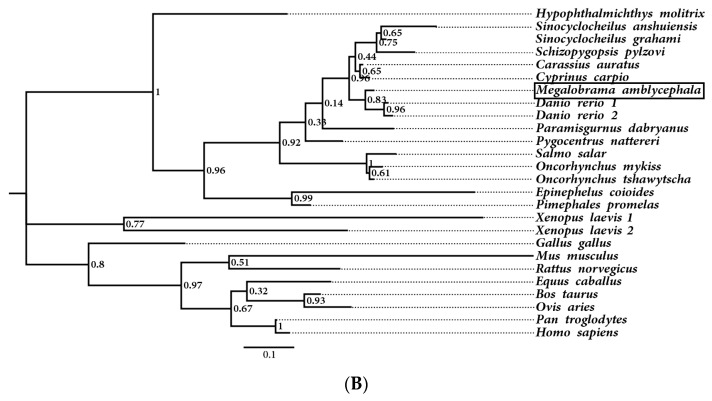
Phylogenetic analysis of vertebrate *hemoglobin α* (**A**) and *β* (**B**) subunits. The accession numbers for these cDNA sequences are listed in Supplementary Tables S2 and S3.

Multiple sequence alignments of the hemoglobin homologs from *M. amblycephala*, *Danio rerio*, *Ictalurus punctatus*, *Salmo salar*, *Homo sapiens*, and *Rattus norvegicus* were performed. The MaHb proteins were highly homologous to hemoglobins from other fish, with a typical globin-like domain, and most of the heme-binding sites and tetramer interfaces (polypeptide-binding sites) were highly conserved among teleosts (Figure 2), indicating fish hemoglobins have maintained their biological functions during evolution. Phylogenetic analysis showed that the hemoglobin genes from *M. amblycephala* and other cypriniformes clustered into one branch, and those from other fish and mammals clustered into other branches (Figure 3), which also revealed the evolutionary conservation of fish hemoglobins.

### 3.3. Expression Patterns of MaHbα and MaHbβ mRNA in Different Tissues and Various Developmental Stages

**Figure 4:**
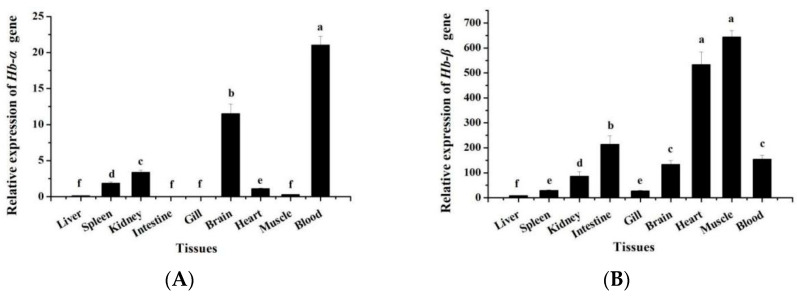
*MaHbα* (**A**) and *MaHbβ* (**B**) expression in various tissues of adult *M. amblycephala*. *18S rRNA* is selected as the reference gene. Data are shown as means ± SE. Different letters above columns indicate statistical significance (*p* < 0.05).

The expression patterns of *MaHbα* and *MaHb*β mRNA in various tissues were elucidated, revealing that *MaHbα* and *MaHbβ* exhibit different tissue distribution patterns. Unlike *MaHbβ*, *MaHbα* is expressed in few tissues, primarily the blood, brain, kidneys, and spleen, followed by the heart and muscle, with marginal expression in the liver, intestine, and gills (Figure 4A). As shown in Figure 4B, *MaHbβ* is widely expressed in *M. amblycephala* tissues. It is strongly expressed in the muscle and heart, followed by the intestine, blood, brain, and kidneys, with low expression in the spleen, gills, and liver (Figure 4B). 

**Figure 5:**
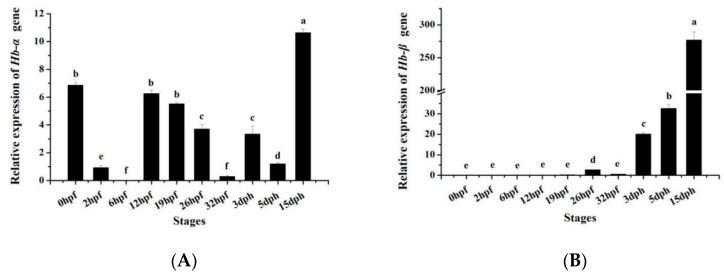
*MaHbα* (**A**) and *MaHbβ* (**B**) expression at different stages of *M. amblycephala* development. *18S rRNA* is selected as the reference gene. Data are shown as means ± SE. Different letters above columns indicate statistical significance (*p* < 0.05).

The *MaHbα* and *MaHbβ* mRNA also exhibited different expression patterns during various developmental stages. As shown in Figure 5A, *MaHbα* expression gradually decreased from 0 hpf to 5 dph with some fluctuation, and then reached its peak level at 15 dph. However, *MaHbβ* has a different mode of expression during early development, showing almost no expression before 26 hpf, then increasing in expression, beginning at 3 dph and reaching extremely high expression at 15 dph (Figure 5B).

### 3.4. MaHbα and MaHbβ Expression in Response to Bacterial Infection

**Figure 6:**
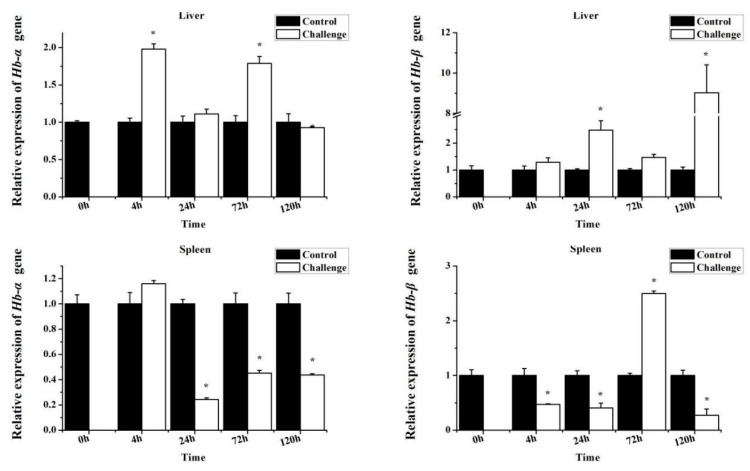

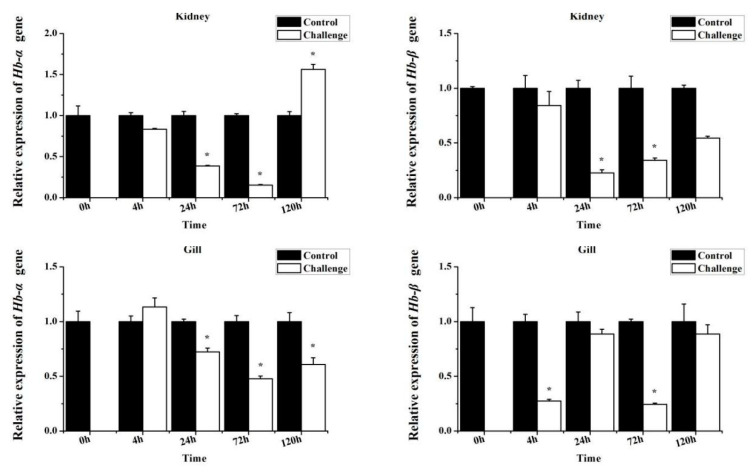
Effect of *A. hydrophila* infection on *MaHbα* and *MaHbβ* mRNA expression. *18S rRNA* is selected as the reference gene. Data are shown as means ± SE. Asterisks (*) above columns indicate statistical significance (*p* < 0.05).

To explore the immune response of *MaHbs* to bacterial infection, juvenile *M. amblycephala* fish were challenged with *A. hydrophila*, and the *MaHbα* and *MaHbβ* mRNA expression levels in the liver, spleen, kidney, and gill tissues were detected using RT-qPCR. In the spleen, kidney, and gills, both *MaHbα* and *MaHbβ* mRNA were significantly down-regulated at most time points within 120 hpi (Figure 6). In the liver, the expression patterns of both *MaHbα* and *MaHbβ* dramatically increased at some time points, unlike the expression patterns in the other tissues (Figure 6).

### 3.5. Antimicrobial Activities of Synthetic MaHbα and MaHbβ Peptides

**Figure 7:**
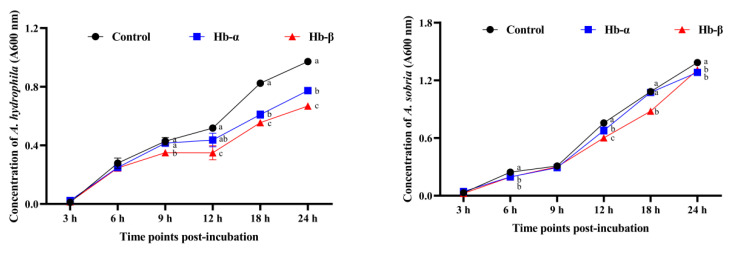

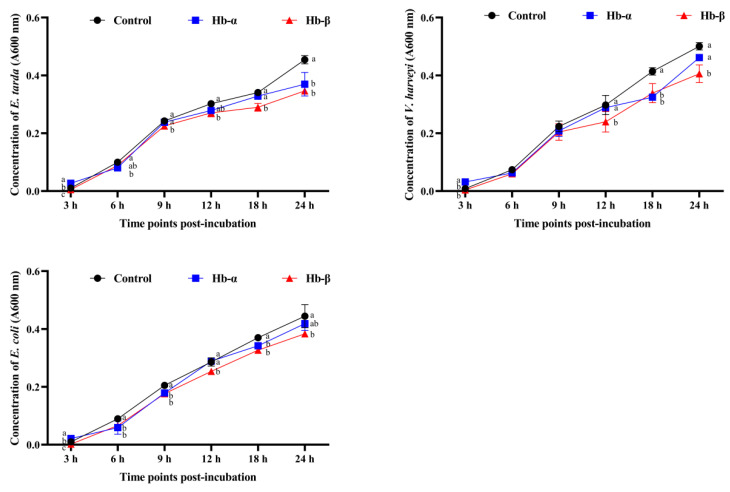
Antimicrobial activities of synthetic MaHbα and MaHbβ peptides against *A. hydrophila*, *A. sobria*, *V. harveyi*, *E. tarda*, and *E. coli*. Data are shown as means ± SE. Different letters above points indicate statistical significance (*p* < 0.05).

The core functional peptides of the MaHbα and MaHbβ proteins, as defined in previous studies, were synthesized. The antimicrobial activities of the synthetic MaHbα and MaHbβ peptides were characterized by assessing their growth inhibition and cytotoxicity toward five aquatic pathogens: *A. hydrophila*, *A. sobria*, *V. harveyi*, *E. tarda*, and *E. coli*. As shown in Figure 7, supplementation of media with the MaHbα and MaHbβ peptides exerted significant effects on bacterial abundance (absorbance at 600 nm), and the bacteriostatic effects displayed exposure time dependence, revealing the efficient antimicrobial activities of the synthetic MaHbα and MaHbβ peptides.

## 4. Discussion

Hemoglobin is an iron-containing allosteric protein found in vertebrate red blood cells. Its main functions including oxygen and carbon dioxide transportation, as well as the maintenance of acid–base balance in the blood. In the present study, we identified and characterized two hemoglobin genes from *M. amblycephala*, termed *MaHbα* and *MaHbβ*. Several hemoglobin subunits from other teleosts, including Atlantic salmon [8], rainbow trout [9], zebrafish [10], carp [11], yellowtail [12], puffer fish [13], medaka [14], and channel catfish [24,25] have been previously characterized. Two *hemoglobin* gene clusters in the fish genome have been demonstrated to constitute hemoglobin tetramers in several fish species. To date, the exact composition of the *M. amblycephala* hemoglobin tetramers remains unknown.

Multiple alignments show that the deduced amino acid sequences of fish hemoglobins are highly conserved, especially vital residues and functional domains. For instance, the Root effect is a specific functional property of fish hemoglobins [26], and *M. amblycephala* hemoglobins retain most of the pivotal residues for the Root effect as its structural basis. Phylogenetic analysis shows that *hemoglobin* genes from *M. amblycephala* and other cypriniformes cluster into one branch, indicating fish *hemoglobin* gene conservation during the evolutionary process.

Previous studies have reported that initial embryonic hemoglobin is involved in primitive hematopoiesis, which occurs in an intermediate cell mass, whereas adult hemoglobin participates in definitive hematopoiesis, mainly in the spleen and kidneys after hatching [14]. Thus, all *hemoglobin* genes from channel catfish are highly expressed in the spleen and kidneys. In the present study, we found that *MaHbα* is mainly expressed in the blood, brain, kidneys, and spleen in *M. amblycephala*, while *MaHbβ* is highly expressed in the muscle, heart, and intestine. The differential expression patterns of these two *M. amblycephala* hemoglobin subunits should reflect their biological functions and suggest that the *MaHbα* subunit is responsible for oxygen storage in the blood vessels and oxygen transport to brain tissue and other oxygen-requiring tissues, but *MaHbβ* may play the oxygen storage and transportation role in the muscle, heart, and intestine. More hemoglobin subunits from *M. amblycephala* need to be identified and genomically characterized to systematically elucidate the composition of gene clusters and expression patterns. 

The expression of zebrafish *hemoglobin* genes exhibits developmental-stage-specific patterns, that is, different subunits can be activated at the embryonic, fetal, and adult stages, respectively [27,28]. For instance, some zebrafish *hemoglobin* subunits are exclusively expressed in mature and adult stages, while others may be exclusively expressed in the embryonic stage or through the embryonic and larval stages [28]. In the present study, *MaHbβ* expression began at 3 dph during development and gradually increased, and was widely expressed in most adult tissues, indicating that *MaHbβ* is synthesized during development and acts as a constitutively expressed subunit. Unlike *MaHbβ*, *MaHbα* expression is detectable and abundant post-fertilization with fluctuating levels during developmental stages, and was detectable in a restricted number of adult tissues, revealing that it likely performs specific functions in the blood and brain.

The expression patterns of *hemoglobin* genes from different aquatic animals upon pathogenic infection are inconsistent. Transcription of two *Scapharca kagoshimensis* hemoglobins (skHbI and skHbII) is significantly up-regulated after *Bacillus subtilis* infection [16], whereas the hemoglobin concentrations of *Cyprinus carpio* and *Carassius auratus* decrease significantly upon *A. hydrophila* infection [29]. In this study, the expression of both *M. amblycephala hemoglobin* subunits was down-regulated in most tissues and time points. This might be due to the invading pathogenic strain of *A. hydrophila* producing virulence factors, such as hemolysin, cytotoxins, or enterotoxins, thereby dissolving red blood cells, damaging capillaries, causing bleeding and sepsis, and reducing the number of host red blood cells [30]. Thereafter, *A. hydrophila* proliferation in the blood to a certain level and circulation of the blood to all parts of the host increase its toxicity and destructiveness, causing damage and dysfunction of the organs, including the kidneys, spleen, and heart [31]. Fish spleen and kidneys are hematopoietic organs; thus, their dysfunction may further reduce the number of red blood cells [32]. Hemoglobin is the main component of red blood cells, and its abundance is positively correlated with the number of red blood cells. Therefore, a reduction in red blood cell number invariably reduces hemoglobin concentration [33]. Thus, reduced *M. amblycephala hemoglobin* genes expression may result from damage to the red blood cells and hematopoietic organs by *A. hydrophila* virulence factors. In addition, the different expression patterns of fish *hemoglobin* genes upon infection might be related to host species, developmental stages, and pathogenic challenge concentrations.

Antimicrobial and bactericidal assays have both shown that chicken hemoglobin antimicrobial peptides (CHAP) exhibit strong and rapid bacteriostatic activities against various bacteria, and electron microscopy (EM) analyses further revealed that CHAP could accumulate many pathogens nearby and quickly penetrate their cell surfaces [34]. Hemoglobin from both horseshoe crabs and humans possesses pseudoperoxidase (POX) activity, which can be synergistically triggered by microbial proteases and pathogen-associated molecular patterns (PAMPs) to produce superoxide anions [35]. Similarly, activation of the POX cycle by bacterial components and stimulated Hb could spontaneously release superoxide radicals in *Ctenopharyngodon idella*, thereby eliciting significant antibacterial properties [7]. Purified *S*. *kagoshimensis* hemoglobins (skHbs) possess excellent antimicrobial activities against Gram-positive bacteria, which might be attributed to their phenoloxidase (PO)-like activities that can detect and kill invading pathogens and synthesize melanin for pathogen encapsulation, thereby involving them in host innate immune responses [15,36]. 

A previous study provides strong evidence that human hemoglobin proteolysis leads to the formation of peptides (β-hemoglobin (111-146) and γ-hemoglobin (130–146)), which effectively inhibit the growth of microorganisms [2]. Minimal inhibitory concentrations of purified bovine α-hemoglobin 107–141, 133–141, and 137–141 peptides clearly reveal the minimal peptide sequence necessary for antibacterial activity to be KYR [37]. Similarly, a bacteriostatic assay detected the minimum inhibitory concentration of β-hemoglobin 114-145 peptide, which showed the minimum antimicrobial peptide to be RYH [38]. In this study, the *M. amblycephala* hemoglobin MaHbα (107–136) and MaHbβ (116–146) peptides were synthesized because they should also be the direct precursors of core antimicrobial peptides [37], which possessed some characteristics of the reported antibacterial peptides [39], containing more than 20 amino acid residues with positive charge, a large number of hydrophobic residues, and a high proportion of α-helical structures. These results show that both synthesized MaHbs peptides exhibited efficient antimicrobial activities against various pathogens, confirming the core antimicrobial peptides were involved in the synthesized regions, while the specific mechanism needs to be further investigated.

## 5. Conclusions

In the present study, *M. amblycephala*’s *hemoglobin α* and *β* subunits were identified and characterized, and their expression patterns and antibacterial activities were elucidated. Multiple sequence alignments and phylogenetic analyses have revealed the evolutionary conservation of fish hemoglobins. The two identified *M. amblycephala Hb* subunits exhibit different expression patterns in various tissues and during development, indicating that they have different physiological functions. In addition, down-regulation of the expression of both subunits after *A. hydrophila* infection may be attributed to damage to the red blood cells and hematopoietic organs. Moreover, the efficient antimicrobial activities of synthesized MaHbα and MaHbβ peptides indicated their immunoprotective effects.

## Figures and Tables

**Table 1:** Primers used in this study.

Primers	Primer Sequences (5′-3′)	Purpose
*MaHbα*-5′-1	ATGCCCACTGGAGGTTTAGCGG	5′-RACE
*MaHbα*-5′-2	GCCGTGCTTCTTCACAGGACCAG	
*MaHbα*-3′-1	CCCTCGGCAGAATGCTGACCGTCTACCCT	3′-RACE
*MaHbα*-3′-2	TCACACAACATCATAGTGGTCATTGGCAT	
*MaHbα*-F	CGGCAGAATGCTGACCGTC	ORF amplification
*MaHbα*-R	GCCCACTGGAGGTTTAGCG	
q*MaHbα*-F	ATGCTCTTCCCTGCTGACTTC	qRT-PCR
q*MaHbα*-R	GGATGCCCACTGGAGGTTTAG	
*MaHbβ*-5′-1	AGCACAACTTTACCGTGAGCAGCAACC	5′-RACE
*MaHbβ-*5′-2	TTGCCAGGGCTTGAGGACCAACGACT	
*MaHbβ*-3′-1	GCAGAAGTTCCTTAGTGTCGTCGTGTCC	3′-RACE
*MaHbβ*-3′-2	CAATGAACACCAGCTGTATTGCAGAAG	
*MaHbβ*-F	CATGGTTGAGTGGACAGACGC	ORF amplification
*MaHbβ*-R	GCGCGGTGCGATCTTCTGC	
q*MaHbβ*-F	GAAACCTCTACAACGCCGC	qRT-PCR
q*MaHbβ*-R	CTTTACCGTGAGCAGCAACC	
q*18S rRNA*-F	CGGAGGTTCGAAGACGATCA	qRT-PCR [18]
q*18S rRNA*-R	GGGTCGGCATCGTTTACG	
q*β-actin*-F	GCTCTTACAGGAAACGGGTC	qRT-PCR [18]
q*β-actin*-R	GCAGCAGCTCTGTAGGTCAT	
q*EF1α*-F	CTTCTCAGGCTGACTGTGC	qRT-PCR [18]
q*EF1α*-R	CCGCTAGCATTACCCTCC	
q*GAPDH*-F	TGCCGGCATCTCCCTCAA	qRT-PCR [18]
q*GAPDH*-R	TCAGCAACACGGTGGCTGTAG	

## Data Availability

The complete cDNA sequences of *MaHbα* and *MaHbβ* genes have been deposited in NCBI database (accession no. KP288030 and OR594269), and other supporting information can be found in Supplementary Materials.

## Supplementary Materials

The following supporting information can be downloaded at: https://www.mdpi.com/article/10.3390/genes14101972/s1, Table S1: Hemoglobin alpha and beta amino acid sequences used for multiple alignment; Table S2: Hemoglobin alpha subunits cDNA sequences used for phylogenetic analysis; Table S3: Hemoglobin beta subunits cDNA sequences used for phylogenetic analysis.
