# Supplementary material for: Characterization and Expression Analysis of Genes from Megalobrama amblycephala Encoding Hemoglobins with Extracellular Microbicidal Activity
Source: Genes (Basel). 2023 Oct 22;14(10):1972. doi: 10.3390/genes14101972 (PMC10606352; doi:10.3390/genes14101972)
Supplement: Supplementary file 1 [file genes-14-01972-s001.zip › genes-2669422-supplementary.pdf]

Supplementary Table S1. Hemoglobin alpha and beta amino acid sequences used for multiple alignment.

Subuni	Species Names	Order	Family	Accession
alpha	*Danio rerio*	Cypriniformes	Cyprinidae	NP_571332.2
	Ictalurus punctatus	Siluriformes	Ictaluridae	NP_001188201.1
	Salmo salar	Salmoniformes	Salmonidae	NP_001117134.1
	Homo sapiens	Primates	Hominidae	NP_000549.1
	Ratus norvegicus	Rodentia	muridae	NP_037228.1
beta	*Danio rerio 1*	Cypriniformes	Cyprinidae	NP_571095.1
	Salmo salar	Salmoniformes	Salmonidae	XP_014042118.1
	Epinephelus coioides	Perciformes	Serranidae	AAK38736.1
	Homo sapiens	Primates	Hominidae	AAR96398.1
	Ratus norvegicus	Rodentia	muridae	NP_942071.1

Supplementary Table S2. *Hemoglobin alpha* subunits cDNA sequences used for phylogenetic analysis.

Species Names	Order	Family	Accession
Carassius auratus	Cypriniformes	Cyprinidae	AM933143.1
Danio rerio	Cypriniformes	Cyprinidae	NM_131257.2
Brycon cephalus	Cypriniformes	Characidae	EF153472.1
Oncorhynchus mykiss	Salmoniformes	Salmonidae	NM_001124551.1
Salmo salar	Salmoniformes	Salmonidae	NM_001123662.1
Esox lucius	Esociformes	Esocidae	BT079758.1
Nibea miichthioides	Perciformes	Sciaenidae	DQ150109.1
Pseudosciaena crocea	Perciformes	Sciaenidae	AY803735.2
Sciaenops ocellatus	Perciformes	Sciaenidae	AY940203.1
Seriola quinqueradiata	Perciformes	Carangidae	AB034640.1
Pagrus major	Perciformes	Sparidae	AY190679.1
Epinephelus coioides	Perciformes	Serranidae	JN112535.1
Treatomus newnesi	Perciformes	Nototheniidae	AY883037.1
Ictalurus punctatus	Siluriformes	Ictaluridae	NM_001201272.1
Anoplopoma fimbria	Scorpaeniformes	Anoplopomatid	BT082697.1
Thunnus thynnus 1	Scombriformes	Scombrida	AB093569.1
Thunnus thynnus 2	Scombriformes	Scombrida	AB093570.1
Xenopus laevis	Anura	Pipidae	NM_001086328.1
Gallus gallus	Galliformes	Phasianidae	NM_001004376.2
Mus musculus	Rodentia	muridae	NM_008218.2
Ratus norvegicus	Rodentia	muridae	NM_013096.1
Canis lupus	Carnivora	Canidae	NM_001270886.1
Pan troglodytes	Primates	Hominidae	NM_001042627.1
Homo sapiens	Primates	Hominidae	NM_000558.3

Supplementary Table S3. *Hemoglobin beta* subunits cDNA sequences used for phylogenetic analysis.

Species Names	Order	Family	Accession
Cyprinus carpio	Cypriniformes	Cyprinidae	XM_019073438.2
Carassius auratus	Cypriniformes	Cyprinidae	AM933144.1
Danio rerio 1	Cypriniformes	Cyprinidae	NM_131020.3
Danio rerio 2	Cypriniformes	Cyprinidae	NM_001005403.1
Hypophthalmichthys	Cypriniformes	Cyprinidae	HM124768.1
Schizopygopsis pylzovi	Cypriniformes	Cyprinidae	KT715803.1
Pimephales promelas	Cypriniformes	Cyprinidae	EF628375.1
Sinocyclocheilus anshuiensis	Cypriniformes	Cyprinidae	XM_016478984.1
Sinocyclocheilus grahami	Cypriniformes	Cyprinidae	XM_016275886.1
Paramisgurnus dabryanus	Cypriniformes	Cobitidae	AF528158.1
Salmo salar	Salmoniformes	Salmonidae	XM_014186643.1
Oncorhynchus mykiss	Salmoniformes	Salmonidae	XM_021625275.2
Oncorhynchus tshawytscha	Salmoniformes	Salmonidae	XM_024390321.2
Pygocentrus nattereri	Characiformes	Serrasalmidae	XM_017718993.2
Epinephelus coioides	Perciformes	Serranidae	AY029506.1
Xenopus laevis 1	Anura	Pipidae	NM_001096347.1
Xenopus laevis 2	Anura	Pipidae	NM_001088028.2
Gallus gallus	Galliformes	Phasianidae	NM_205489.3
Equus caballus	Perissodactyla	Equidae	NM_001164018.1
Bos taurus	Artiodactyla	Bovidae	NM_173917.2
Ovis aries	Artiodactyla	Bovidae	NM_001097648.1
Mus musculus	Rodentia	muridae	NM_001127686.1
Ratus norvegicus	Rodentia	muridae	NM_198776.1
Pan troglodytes	Primates	Hominidae	XM_508242.5
Homo sapiens	Primates	Hominidae	AY509193.1
